# Role of Minimally Invasive (Micro and Ultra-mini) PCNL for Adult Urinary Stone Disease in the Modern Era: Evidence from a Systematic Review

**DOI:** 10.1007/s11934-018-0764-5

**Published:** 2018-03-07

**Authors:** Patrick Jones, Muhammad Elmussareh, Omar M. Aboumarzouk, Phillip Mucksavage, Bhaskar K. Somani

**Affiliations:** 1 Department of Urology, University Hospital Southampton, Southampton, UK; 2 Department of Urology, Pinderfields Hospital, Aberford Rd, Wakefield, UK; 3 Department of Urology, Queen Elizabeth University Hospital, Scotland, UK; 4 Perelman School of Medicine, University of Pennsylvania, Philadelphia, PA USA

**Keywords:** PCNL, Percutaneous nephrolithotomy, Ultra-mini, UMP, Micro, Stone

## Abstract

**Purpose of Review:**

The role of PCNL and the expertise surrounding it has expanded in recent decades. Miniaturisation of equipment and instrument size has formed a part of this innovation. Although an increasing number of studies have been performed on miniaturised PCNL (Mi-PCNL) recently, a critical appraisal on these is lacking. We therefore conducted a systematic review of the literature to evaluate the efficacy, safety and feasibility of Mi-PCNL techniques (< 15 Fr).

**Recent Findings:**

A systematic review was conducted from 1990 to March 2017 on outcomes of Mi-PCNL [micro PCNL (m-PCNL) and ultra-mini PCNL (UMP)] in adult patients. Ten studies (three on m-PCNL and seven on UMP) were included in our study. Across the three studies, 118 patients (mean age 42.2 years, male to female ratio 1.3:1) underwent m-PCNL (4.8 Fr). For a mean stone size of 13.9 mm, a mean stone-free rate (SFR) was 89% and an overall complication rate was 15.2% [Clavien classification I (44%), II (28%), III (28%)], with no Clavien IV or V complications. Across the seven studies, 262 patients (mean age 49.4 years, male to female ratio 1.5:1) underwent UMP (13–14 Fr). For a mean stone size of 18.6 mm, a mean SFR was 88.3% and an overall complication rate was 6.2% [Clavien classification I (57%), II (36%), III (7%)], with no Clavien IV or V complications. While the transfusion rates for m-PCNL was 0.85%, only one case each in m-PCNL and UMP needed conversion to mini PCNL.

**Summary:**

Our review shows that for small- to medium-sized renal stones, Mi-PCNL can yield good stone-free rates whilst maintaining a low morbidity associated with it. There were no Clavien > III complications and no mortality with only one transfusion reported from this minimally invasive technique.

## Introduction

Percutaneous nephrolithotomy (PCNL) was described almost 40 years ago, and since then, it has continued to undergo innovation and minimization [1]. These advancements have been largely focussed towards delivering greater stone clearance, while minimising morbidity, procedure time and length of stay. As such, the therapeutic potential for PCNL has expanded alongside retrograde intrarenal surgery (RIRS) and shockwave lithotripsy (SWL) [2•].

Given the recognised risks of haemorrhage and organ injury associated with tract creation and dilation in standard PCNL, a key strategy aimed at reducing this has been the miniaturisation of equipment/instrument size, as well as the introduction of laser technology and improved optic systems. In recent years, an increasing number of studies have been performed, reporting their experiences and results with miniaturised PCNL (Mi-PCNL) techniques (micro and ultra-mini). However, critical appraisal on these remains lacking. Therefore, we aimed to conduct a systematic review of the literature and evaluate the efficacy, safety and feasibility of Mi-PCNL techniques.

## Methods

### Search

A search strategy was formulated by the authors and applied to the following databases: Pubmed, Web of Science, ScienceDirect, CINAHL, Scopus and the Cochrane Library (search conducted in March 2017). This was carried out in a Cochrane style format and in accordance with the Preferred Reporting Items for Systematic reviews and Meta-Analyses (PRISMA) checklist.

The aim was to identify all original studies relating to micro (m-PCNL) and ultra-mini (UMP) PCNL published between 1990 and March 2017. Citation lists of relevant articles as well as conference abstracts were also hand searched.

Search terms included “percutaneous nephrolithotomy”, “PNL”, “PCNL”, “mini”, “ultra-mini”, “micro”, “UMP”, “kidney stones” and “urolithiasis”. Medical subject headings (MeSH) included [Urologic Surgical Procedures], [Minimally Invasive Surgery], [Nephrolithiasis], and [Urolithiasis].

### Inclusion/Exclusion Criteria

The search did not carry any time or language restrictions. All study types were considered from case series to randomised trials. Animal studies and lab-based studies were excluded. Only studies with at least five patients in their sample size were included in order to gather results from centres with sufficient endourological experience in these new techniques. Studies performing PCNL with instruments ≥ 15 Fr were excluded. The identified studies were categorised into two groups according to instrument size employed: ultra-mini PCNL (11–14 Fr) and micro PCNL (< 10 Fr). The largest instrument size that was used in individual studies has been recorded in this review. Studies were required to report on adult (≥ 18 years) patients only. Articles reporting on both adult and paediatric patients were excluded unless we could retrieve data for adult patients from these studies. For multiple studies reporting from the same core data set, the most recent or comprehensive article was selected for inclusion.

### Data Extraction and Outcomes of Interest

Two authors performed the literature search and data extraction independently (PJ and ME.) and any disparity was agreed after arbitration from the senior author (BKS). Primary outcomes of interest were stone-free rate (SFR), complications recorded and transfusion rates. Secondary outcomes of interest included length of stay and operative time. Additional information was collected on baseline demographics, stone size, Hounsfield units (HU), instrument size and post-operative drainage (nephrostomy or JJ stent insertion).

Each study was assigned a level of evidence in accordance with the criteria set by the Centre for Evidence-Based Medicine (CEBM) [3]. Given the heterogeneity of results, formal meta-analysis was precluded and therefore pooled analysis of mean results and narrative description has been carried out.

## Results

From a total of 607 studies, 10 were identified which satisfied our pre-defined selection criteria for Mi-PCNL [4••, 5–11, 12•, 13]. Three of these studies related to micro PCNL (two retrospective studies and one randomised study, published between 2013 and 2015) [4••, 5, 6]. The seven remaining articles all described ultra-mini PCNL, also termed UMP (one case series, four cohort studies and two randomised studies, published between 1998 and 2016) [7–11, 12•, 13] (Table 1).

**Table 1:** Baseline characteristics of the reported studies

Author	Year	Country	Study type/level of evidence	Number patients	Mean age (SD, range)	Male/female	Mean BMI (SD)	Instrument size
Micro PCNL								
Sabnis [4••]	2013	India	Single-centre, prospective, randomised study	35	38.6 (14.6,NR)	22/13	23.9 (4.9)	4.8
Bagcioglu [5]	2015	Turkey	Single-centre, retrospective, cohort study	63	41.5 (13.9, NR)	34:29	29.3 (4.1)	4.8
Olcucuoglu [6]	2015	Turkey	Single-centre, retrospective, cohort study	20	46.5 (13.8, NR)	11/9	NR	4.8
Ultra-mini PCNL								
Jackman [7]	1998	USA	Single-centre, retrospective, case series	7	57 (NR,43–73)	NR	NR	13
Schoenthaler [8]	2015	Germany/UK	Multicentre, retrospective, cohort study	30	54.3 (NR, 19–72)	17/13	29.9 (NR, 18.7–42.1)	14
Shah [9]	2015	China	Single-centre, retrospective, cohort study	22	49.1 (11.2, 22–73)	14/8	25.1 (3.2, 21.9–29.3)	13
Pullar [10]	2016	UK	Multicentre, retrospective, cohort study	32	52 (NR,21–79)	16/16	NR	13
Demirbas [11]	2016	India	Single-centre, prospective, randomised study	30	43.73 (14.62, NR)	21/9	NR	14
Datta [12•]	2016	India	Single-centre, prospective, cohort study	94	46.5 (13.7,NR)	58/36	NR	13
Karakan [13]	2016	Turkey	Single-centre, prospective, randomised study	47	43.3 (NR, 19–69)	28/19	29 (5.1, NR)	14

*NR* not reported

### Micro PCNL in Adults

Across the three studies, 118 patients (mean age 42.2 years, male to female ratio 1.3:1) underwent micro PCNL (4.8 Fr) [4••, 5, 6]. Two thirds of studies reported the average BMI (range 23.9–29.3 kg/m^2^). The mean stone size was 13.9 mm (range11–17.7 mm), with a mean SFR of 89.3% (range 80.9–97.1%). In 46/118 patients, a JJ stent was placed intra-operatively (Table 2). The mean operative time and length of stay was 87 min (range 20–150 min) and 2.2 days (range 1–3 days) respectively. Breakdown by stone location was as follows: Lower pole (49.6%), renal pelvis (27.7%), middle pole (15.5%) and upper pole (7.2%); however, none of the studies provided information on stone composition (Table 3).

**Table 2:** Results of the reported studies

Author	Mean operative time (SD, range)		Mean hospital stay (SD, range)—days		Mean stone size (SD)	Mean Hounsfields units (SD, range) HU	Stone-free rate (%)	Definition of stone free (imaging modality)	JJ stenting (%)	Complications	Mean Hb drop (g/L)		Blood transfusion (%)
Micro PCNL													
Sabnis [4••]	51.6 (18.5,20–90)		2.4 (0.9,NR)		1.1 (0.23) cm	1313 (203,NR)	97.1	NR (XR)	20	Haematuria (5), fever (3), minor pelvic perforation (1)	9.6		0
Bagcioglu [5]	98.5 (29.64,NR)		2.72 (1.58,NR)		1.77 (0.56,NR)	NR	80.9	NR	57	Colic pain (2), steinstrasse (5)	9.1		0
Olcucuoglu [6]	111 (37,NR)		1.4 (0.8,NR)		13 (3,NR)	NR	90	≤ 4 mm (XR,USS or NCCT)	15	Urinary infection (1), haemorrhage requiring blood transfusion (1)	12		5
Ultra-mini PCNL													
Jackman [7]	176 (123–270		1.7 (NR,1–3)		1.5 (NR) cm^2^	NR	89	NR (XR)	100	NR	NR		0
Schoenthaler [8]	121 (NR,79–170)		2.3 (NR,1–6)		15.1 mm (NR,10–20)	NR	84	NR	0	Complication rate 7%. No further details given	NR		NR
Shah [9]	85.7 (18, 47–112)		3.1 (1.8, 2–5)		26.6 (4.7) mm	NR	81.8	≤ 4 mm (XR)	100	Fever (1), Haematuria resolving spontaneously (2)	12		0
Pullar [10]	103 (NR, 49–150)		1.1			1123 (NR, 404–1662)	96	NR (XR or NCCT)	16	Perinephric haematoma (1), colic pain (1)	11		0
Demirbas [11]	54.53 (23.09)		2.46 (3.02, NR)		185.86 (88.29,NR) mm^2^	NR	80	< 3 mm (NCCT)	NR	Clavien I-II: 2, Clavien IIIA/B: 5. No further details	NR		NR
Datta [12•]	Stone < 20 mm	Stone 21–30 mm	Stone < 20 mm	Stone 21–30 mm	15.91 (4.5) mm	1106 (167)	98	NR 2), (NCCT)	NR	Haemorrhage resolving spontaneously (2), fever (2), perinephric collection requiring drainage (1)	Stone < 20 mm	Stone 21–30 mm	0
	51.8 (NR,NR)	63.9 (NR,NR)	1.6 (NR,NR)	1.7 (NR,NR)							7.6	11.5	
Karakan	55 (23.5,NR)		1 (NR,1–4)		20.3 (3) mm	NR	89.3	NR	6.3	Colic pain (2), fever (1), urinary infection (1),	NR		0

*NR* not reported

**Table 3:** Stone characteristics (site and composition of stone)

Author	Site of stone							Stone composition							
	Upper pole %	Middle pole %	Lower pole %	Renal pelvis %	PUJ/upper ureter %	Multi-calyceal %	Calyceal diverticulum %	Calcium oxalate %	Calcium phosphate %	Mixed calcium oxalate/phosphate %	Uric acid %	Mixed calcium oxalate/uric acid	Cystine %	Brushite %	Unknown or other %
Micro PCNL															
Sabnis [4••]	8.6	8.6	42.8	40	–	–	–	NR	NR	NR	NR	NR	NR	NR	NR
Bagcioglu [5]	13	13	31	43	–	–	–	NR	NR	NR	NR	NR	NR	NR	NR
Olcucuoglu [6]	–	25	75	–	–	–	–	NR	NR	NR	NR	NR	NR	NR	NR
Ultra-mini PCNL															
Jackman [7]	NR	NR	NR	NR	NR	NR	NR	NR	NR	NR	NR	NR	NR	NR	NR
Schoenthaler [8]	NR	NR	NR	NR	NR	NR	NR	NR	NR	NR	NR	NR	NR	NR	NR
Shah [9]	4.5	4.5	13.5	55	9	13.5	–	13.5	9	22.5	4.5	–	–	–	51.5
Pullar [10]	6.3	–	53.1	31.2	6.3	3.1	–	41	–	16	9	3	3	3	25
Demirbas [11]	–	–	50	40	–	10	–	NR	NR	NR	NR	NR	NR	NR	NR
Datta [12•]	7	5	30	55	2	–	1	Majority of stones were calcium oxalate. No further details given.							
Karakan [13]	21.2		29.7	40.4	–	8.5	–	NR	NR	NR	NR	NR	NR	NR	NR

*NR* not reported

### Complications of m-PCNL

The overall complication rate was 15.2% [Clavien classification I (44%), II (28%), III (28%)], with no Clavien IV or V complications reported (Table 3). While the commonest minor complication reported was haematuria that resolved spontaneously (*n* = 5), all Clavien III complications were steinstrasse requiring emergency JJ stent insertion. While the mean drop in Hb was 10.2 g/L, the overall transfusion rate was 0.85% and only one case required conversion to mini PCNL.

### Ultra-mini PCNL in Adults

Across the seven studies, 262 patients (mean age 49.4 years, male to female ratio 1.5:1) underwent UMP (13–14 Fr) [7–11, 12•, 13] (Table 1). Three studies included information on patients’ BMI (range 25.1–29.9 kg/m^2^). The mean stone size was 18.6 mm (range 10–25 mm), with a mean SFR of 88.3% (range 81.8–98%) (Table 2). Pullar et al. provided no mean value but operated on a range of stones sized from 5 to 24 mm [10]. Five studies reported on placement of JJ stents intra-operatively (mean 44.5%, range 0–100%). Mean operative time and hospital stay was 88.9 min (range 50–270 min) and 1.8 days (range 1–6 days) respectively. Breakdown of stone location was as follows: renal pelvis (44.3%), lower pole (35.3%), multi-calyceal (7%), upper pole (5.7%), middle pole (4%) and calyceal diverticulum (0.2%) (Table 3). Two studies provided details on stone composition, which revealed calcium oxalate (27.3%) and mixed calcium oxalate/phosphate (19.3%) as the commonest compositions (Table 3).

### Complications of UMP

Five of the reported studies provided full breakdown of complications (Table 3). Of these, the overall complication rate was 6.2% [Clavien classification I (57%), II (36%), III (7%)], with no Clavien IV or V complications in any of these studies. Amongst the remaining two studies, Schoenthaler et al. reported a complication rate of 7% [8] and Demirbas et al. reported a complication rate of 23.4% including two (6.7%) Clavien I-II complications and five (16.7%) Clavien III complications [11]. Neither of these studies gave any further details. While the mean drop in Hb across all studies was 10.5 g/L, none of them reported any blood transfusion and no deaths were reported in any of them. In total, only one case (0.4%) had to be converted to standard PCNL.

## Discussion

### Findings of Our Study

Our systematic review is the first paper reporting on outcomes of micro and ultra-mini PCNL in adult patients. There has been a marked rise in original studies published on this topic, likely reflecting the increasing benefits associated with the miniaturised-PCNL techniques. The outcomes reflect a good SFR with small risk of minor (Clavien I-III) complications in adult patients. Even in studies reporting on mixed paediatric and adult patients, the results were not too dissimilar with excellent outcomes. Our findings are however limited to the quality of these studies most of which were retrospective in origin and might have a selection bias associated with them.

### Advantages of Miniaturised PCNL

Use of Mi-PCNL is applicable for renal stones in cases where the kidney cannot be accessed retrogradely via the ureter (difficult or tight ureter or in cases of urinary diversion). This is an advantage over retrograde intrarenal surgery (RIRS) where the failed access rate can be up to 15%, thereby also avoiding a risk of ureteric injury which is not uncommon either on its own or with the use of an access sheath [14]. With a rising use of Mi-PCNL, there is also a proportionate decrease in the use of post-operative ureteric drainage with JJ stent insertion, with studies now reporting an increase in the totally tubeless (TT) procedures. Similarly, increasingly a ureteral catheter can be placed and removed 24–48 h post operatively.

In contrast, in a recent multicentre Clinical Research Office of the Endourology Society (CROES) study, use of Double J stenting post RIRS was 88% [15]. Stent-related symptoms are well recognised and potentially Mi-PCNL can avoid this as well as the subsequent visits to department necessary for their removal. Pullar et al. were able to discharge 88% of their patients on day 1 post procedure [10].

The overall complication rate in the British Association of Urological Surgeons (BAUS) PCNL registry and CROES PCNL registry, was 21.3% and it was 20.5 respectively [16, 17]. This study found the overall complication rate to be more favourable than those for standard PCNL (m-PCNL 15.2%, UMP 6.2%). These were predominantly low grade in nature and no Clavien IV/V complications were recorded. The optic needle PCNL access theoretically allows for avoidance of injury to the renal parenchyma and other viscera. This method also permits a single step process for renal access and therefore reduces time to initiating lithotripsy [18]. Desai et al. also advocate its use for a safer anterior calyx and supra-costal puncture [19]. The therapeutic application of micro and UMP extends to the paediatric population [18, 20, 21]. Liu et al. reported on 111 infants (mean age 3.9 years) who underwent UMP. Initial and final SFR was 84.7% (94/111) and 90.1% (100/111) respectively [21]. Of the cases, 85.6% were successfully performed completely tubeless. The advantage of a single session Mi-PCNL technique compared to staged RIRS needs to be weighted, especially in the paediatric age group which necessitates a further general anaesthetic and the psychological disturbance that can be caused by a repeat hospital admission and surgical procedures. For those experienced with standard PCNL, the transition to use of smaller instruments is a step in the right direction especially for stones between 1 and 2.5 cm and studies suggest a better intra-operative surgeon comfort using Mi-PCNL techniques [22].

### Disadvantages of Mi-PCNL

Use of Mi-PCNL equipment leads to longer operative times to achieve fragments of a size small enough to fit through the smaller instrument channel. Furthermore, m-PCNL does not allow for stone extraction but relies on passive clearance of fragments similar to SWL. In this respect, stone analysis can be difficult (unless sieved by the patient during urination). This inability to retrieve fragments leads to a higher rate of steinstrasse, therefore necessitating urgent drainage of the collecting system via JJ stenting.

### Cost and Areas of Future Research

Overall, there is a paucity of research addressing the economic feasibility of miniaturised techniques. Given the current pressures across all healthcare systems, the financial implications are of ever-growing importance. Schoenthaler et al. did however compare costs for UMP and RIRS [8]. Their cost analysis revealed total average cost per case of 656 euro (UMP) and 1160 euro (RIRS), which includes price of disposables and endoscopes. Bagcioglu et al. compared cost of RIRS and micro PCNL and also found significant cost saving with the latter technique per case ($917.13 versus $831.58*, p* < 0.001) [5].

A further advantage of Mi-PCNL methods is therefore the cost benefits for the health care provider. This is largely related to the higher use of re-usable equipment, durability associated with these techniques and the cost associated with the purchase, as opposed to the maintenance of ureteroscopes with the additional cost of disposables for individual patients with RIRS [23••].

The definition of stone-free rate (SFR) also need to be standardised so that it is easier to compare the outcomes of different treatment modalities [24]. Similarly, apart from surgical and outcome research, more also needs to be done in identifying factors associated with, and for prevention of kidney stone disease [25, 26].

## Conclusion

While standard PCNL and RIRS remain the benchmark surgical interventions for urinary stone disease, the potential role for miniaturised PCNL techniques is emerging. This article has found that for small- to medium-sized renal stones, it can yield good stone-free rates whilst maintaining a low morbidity associated with it. Its use is perhaps arguably most useful for lower pole stones and where SWL and RIRS have a higher tendency to fail. Although with time its formal role will become clearer, further randomised trials performed in the multicentre setting are vital to achieve this.

## Abbreviations

Mi-PCNL: Miniaturised PCNL
m-PCNL: Micro PCNL
UMP: Ultra-mini PCNL
SWL: Shockwave lithotripsy
URS: Ureteroscopy
PCNL: Percutaneous nephrolithotomy
